# Integrative analyses of metabolome and transcriptome reveal the dynamic accumulation and regulatory network in rhizomes and fruits of *Polygonatum cyrtonema* Hua

**DOI:** 10.1186/s12864-024-10608-4

**Published:** 2024-07-19

**Authors:** Luyun Ning, Yuanshu Xu, Lu Luo, Limin Gong, Yeman Liu, Zhi Wang, Wei Wang

**Affiliations:** 1 TCM and Ethnomedicine Innovation and Development International Laboratory, Innovative Material Medical Research Institute, School of Pharmacy, Hunan University of Chinese Medicine, Changsha, China; 2 School of Pharmacy, Hunan University of Chinese Medicine, Changsha, China

**Keywords:** *Polygonatum cyrtonema*, Rhizomes, Fruits, Metabolome, Transcriptome

## Abstract

**Background:**

According to Chinese ancient books, both fruits and rhizomes of *Polygonatum cyrtonema* Hua have medicinal and edible values. Up to now, there is no report about the metabolite profiles and regulatory network in fruits and different year-old rhizomes of *P. cyrtonema*.

**Results:**

In this study, we performed integrative analyses of metabolome and transcriptome to reveal the dynamic accumulation and regulatory network of fruits and different year-old rhizomes in *P. cyrtonema*. The relative content of phenolic acids, lignans and coumarins, flavonoids and alkaloids increased with growth years, while steroids and lipids decreased with it. In addition, the relative content of nucleotides and derivatives, flavonoids, organic acids, steroids and lipids in fruits were higher than rhizomes. Genes that might relate to the biosynthesis of polysaccharides, flavonoids, triterpene saponins and alkaloids biosynthesis were further analyzed by transcriptome analysis, including *sacA*, *GMPP*, *PMM*, *CCoAOMT*, *CHI*, *ANR*, *CHS*, *DXS*, *GGPS*, *ZEP*, *CYP72A219* and so on, for their expressions were positively correlated with the relative content of the metabolites. Additionally, the correlation network in sugar and aromatic amino acids metabolites were constructed to further illustrate the biosynthesis of polysaccharides, flavonoids and alkaloids in *P. cyrtonema*, and some transcription factors (TFs) were screened, such as *C2C2*, *MYB*, *bZIP*, *GRAS* and *NAC*.

**Conclusions:**

This study can deepen our understanding of the accumulation patterns and molecular mechanism of the main compounds in *P. cyrtonema*, and provide reference for the standardize production of *P. cyrtonema*.

**Supplementary Information:**

The online version contains supplementary material available at 10.1186/s12864-024-10608-4.

## Background

*Polygonatum cyrtonema* Hua (also known as Huangjing in China) is a perennial herb combining medicinal and edible values, and has broad prospects for development and utilization. According to Chinese Pharmacopoeia, the dried rhizomes of *P. cyrtonema* are used as the main medicinal part. Additionally, the seedling, leaf, flower and fruit of *P. cyrtonema* can also be used as food or medicine in terms of our Chinese ancient books, and the flower and fruit had more excellent value than rhizome [1]. The yield of fruits was much bigger than flowers, so fruits might be a potential tissue with the value of development and utilization in *P. cyrtonema*.

To date, some bioactive compounds have been extracted from *P. cyrtonema*, such as polysaccharides, flavonoids, steroidal saponins, triterpene saponins and alkaloids; the extensive pharmacological activities have also been identified, such as cytotoxic, antioxidant, anti-fatigue and anti-inflammatory activities [2, 3]. Particularly, the content of total polysaccharides was regarded as the quality standard of *P. cyrtonema* according to Chinese Pharmacopoeia. Nowadays, the edible and medicinal value of *P. cyrtonema* is well known, and the leisure food and health product made of *P. cyrtonema* rhizomes are very popular among the public in China. The development of health products of *P. cyrtonema* were mainly preliminarily processed product, such as nine steaming and nine processing Huangjing, Huangjing pills, Huangjing wine, Huangjing drink, Huangjing cookies and Huangjing yogurt [4, 5]. In addition, the deep-processing products were also explored, for example, sweetened roll of *rhizoma polygonati*, hawthorn and yam, which combined the characteristics of sweet tasty, improving memory and anti-aging [6]. So, it’s of great importance to standardize the growth and production of *P. cyrtonema* to guarantee quality of the products and the scientific nature of clinical medication.

**Fig. 1:**
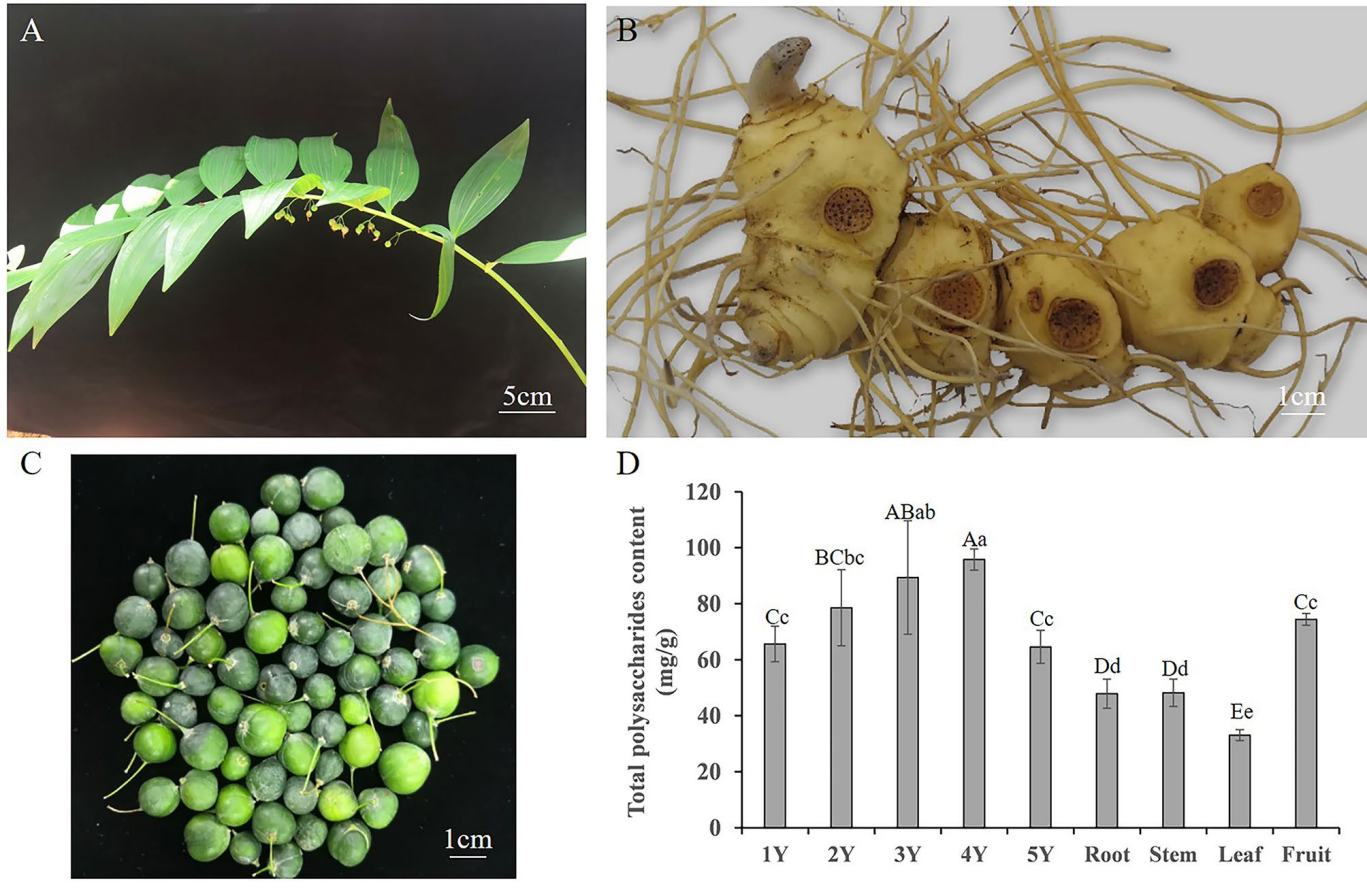
The total polysaccharides content in different tissues of *P. cyrtonema*. **A** The overground part of *P. cyrtonema*. **B** The rhizomes of *P. cyrtonema*. The left node with buds was the 1-year-old (1Y) node of rhizome, then was the 2-year-old (2Y) node of rhizome, 3-year-old (3Y) node of rhizome, 4-year-old (4Y) node of rhizome and 5-year-old (5Y) node of rhizome successively. **C** The fruits of *P. cyrtonema*. **D** The total polysaccharides content in 1Y, 2Y, 3Y, 4Y and 5Y rhizome, root, stem, leaf and fruit of *P. cyrtonema*. mg/g on Y-axis meant content in dry weigh. The lowercase letters and capital letters indicate the significance difference at *P* < 0.05 and *P* < 0.01, respectively. (mean ± SD, *n* ≥ 3)

Generally speaking, the underground rhizome of *P. cyrtonema* elongate only one node in a year [7], for example, the rhizome of 5-year-old *P. cyrtonema* only has 5 nodes (Fig. 1A and B). Previous study has shown that, the content of both total polysaccharides and total saponins in *P. cyrtonema* rhizomes increased with the growth years [8]. What’s more, after performing the nine steaming and nine processing on *P. cyrtonema* rhizomes, the changes of mineral elements were different in each node [9]. Different age of medical parts had different level of compounds, which was universal in medical plants, for example, *Panax ginseng*. The solid content of 5-year-old and 6-year-old ginseng was significantly higher than that of 3-year-old and 4-year-old ginseng [10]. Additionally, the untargeted metabolomic analysis divided ginseng samples aged 4–20 years into 3 age groups clearly, and 22 potential age-dependent biomarkers were screened to differentiate the 3 sample groups [11]. Hence, systematically exploring the content of components in different nodes of *P. cyrtonema* rhizome was necessary.

Up to now, the breeding research of *P. cyrtonema* is relatively stagnant, though success in its manual cultivation and expansion of large-scale planting in recent years. In general, *P. cyrtonema* produce flowers and fruits at the fourth year after seeding [12], so it will be very difficult and time-consuming by traditional breeding. Nowadays, molecular breeding is very popular in both crops and medicinal plants, for the advantage of fast, efficient and accurate [13]. Multi-omics, including genomics, transcriptomics, proteomics, metabolomics, phenomics and so on, can provide the convenient of selecting key genes for molecular breeding [14–16]. Hence, molecular breeding will be a potential convenient method to improve the quality of *P. cyrtonema*.

To date, some omics studies have been reported in *P. cyrtonema*. RNA-seq of different tissues and different year-old rhizomes were preformed to identify genes related to polysaccharide and saponin biosynthesis [8, 17]. RNA-seq and micro-RNA analyses of different tissues were conducted to uncover the accumulating mechanisms in *P. cyrtonema* [18]. Recently, the UHPLC-Q-TOF-MS untargeted metabolomics has been used to verify the metabolite profiles in diverse *Polygonati* rhizomes and identified 1126 metabolites [19]. Moreover, 22 flavonoids were identified by using widely-targeted metabolome analysis from 15 genotypes of *P. cyrtonema* [20]. However, these works were not enough to illustrate the biosynthesis pathways and accumulation patterns of the main compounds in *P. cyrtonema*.

In the present study, UPLC–MS/MS-based widely targeted metabolomics analysis was conducted to illustrate the compounds in fruits and different year-old rhizomes, and RNA-seq of these tissues was also performed to select the key genes. This study deepened our understanding of the accumulation patterns and biosynthesis molecular mechanism of the main compounds in *P. cyrtonema*, provided reference for the standardize production and screened the potential candidate regulatory factors for molecular breeding in *P. cyrtonema*.

## Results

### Total polysaccharides content in different tissues of *P. cyrtonema*

Total polysaccharides were extracted and detected in different tissues (Fig. 1). The total polysaccharides content increased with the growth years at first, the value was higher in 3Y (89.3 mg/g) and 4Y rhizomes (95.8 mg/g), while significantly decreased in 5Y rhizomes (64.5 mg/g) (Fig. 1D). Interestingly, the total polysaccharides content in fruits was nearly the same as 1Y and 5Y rhizomes. The lowest total polysaccharides content appeared in leaves (33.0 mg/g).

### Comparative metabonomic analysis in fruits and different year rhizomes of *P. cyrtonema*

**Fig. 2:**
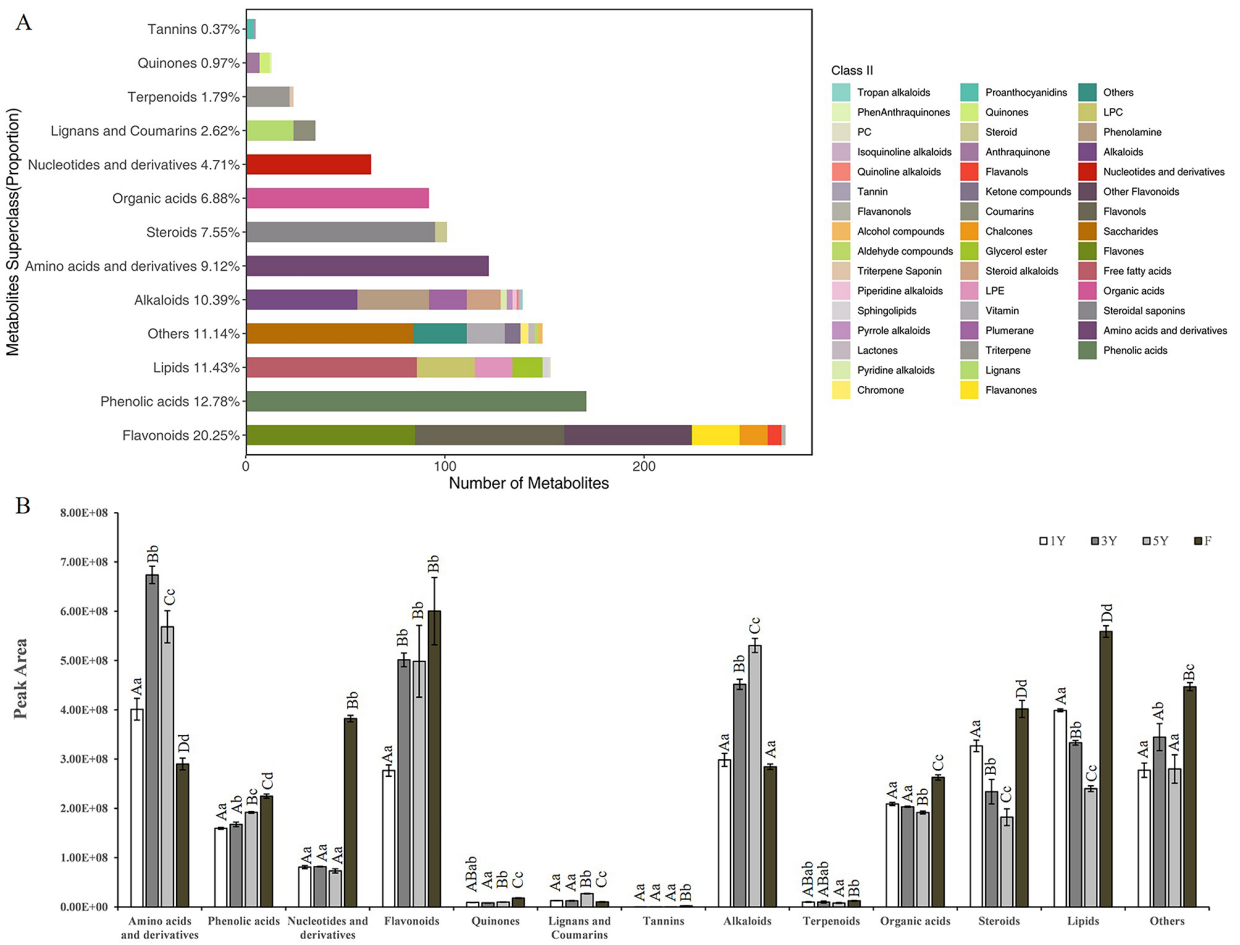
The identified metabolites classification histogram and the expression of different categories in fruits and different year-old rhizomes of *P. cyrtonema*. **A** Identified metabolites classification histogram. **B** Peak area of different categories in fruits and different year-old rhizomes of *P. cyrtonema*. The lowercase letters and capital letters indicate the significance difference at *P* < 0.05 and *P* < 0.01, respectively. (mean ± SD, *n* = 3)

A total of 1338 metabolites (divided into 13 categories) were detected and quantified (Fig. 2A; Table S2), including 271 flavonoids (20.25%), 171 phenolic acids (12.78%), 153 lipids (11.43%), 149 others (11.14%, including saccharides, vitamin, ketone compounds and so on), 139 alkaloids (10.39%), 122 amino acids and derivatives (9.12%), 101 steroids (7.55%), 92 organic acids (6.88%), 63 nucleotides and derivatives (4.71%), 35 lignans and coumarins (2.62%), 24 terpenoids (1.79%), 13 quinones (0.97%) and 5 tannins (0.37%). The 13 categories of metabolites in different samples were shown in Fig. 2B (Table S3), which indicated that the relative content of phenolic acids, flavonoids, lignans and coumarins and alkaloids were increased with the rhizomes’ growth year. In addition, the relative content of steroids and lipids were decreased with the rhizomes’ growth year. Interestingly, the relative content of nucleotides and derivatives, flavonoids, organic acids, steroids and lipids were higher in the fruits. PCA results showed that PC1 and PC2 explained 44.66% and 21.07% of the total variance, respectively (Fig. S1A). 5Y rhizomes and fruits were distinguished from 1Y and 3Y rhizomes by PC1 and PC2. Hierarchical Cluster analysis (HCA) (Fig. S1B) indicated that according to the relative abundance of all annotated metabolites, the concentrations of most detected metabolites among different samples were significantly different, which further confirmed the PCA results. Moreover, the biological replicates in each group showed a high correlation (Fig. S1C).

**Fig. 3:**
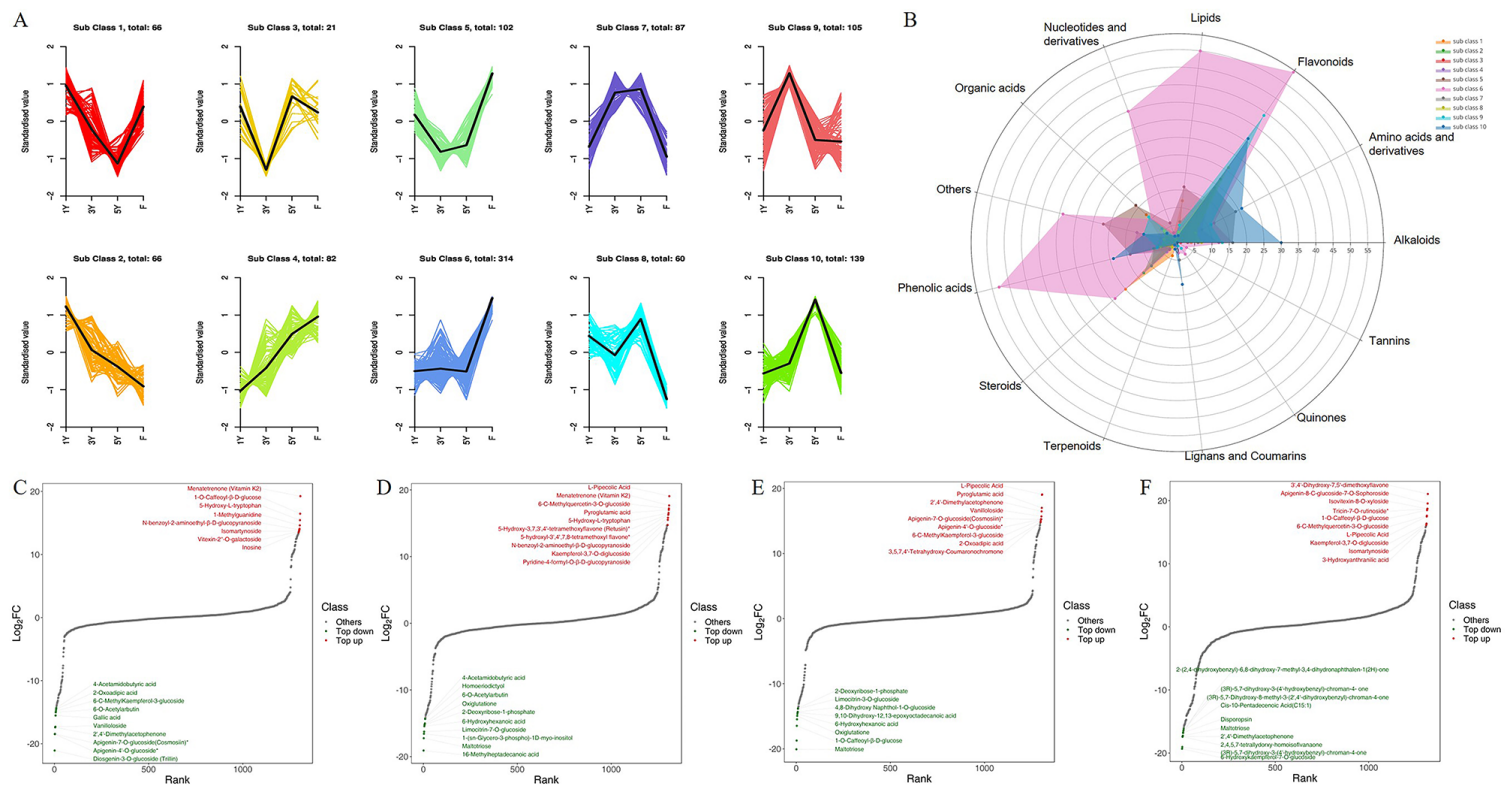
Analysis of dynamical changes of DAMs. **(A)** K-means cluster analysis of DAMs. **(B)** The number of different types of DAMs in different subclasses. The top ten up- and down-regulated DAMs in 1Y vs. 3Y **(C)**, 1Y vs. 5Y **(D)**, 3Y vs. 5Y **(E)** and 1Y vs. F **(F)** are shown

Compared with 1Y rhizomes, 228 up-regulated and 199 down-regulated differentially accumulated metabolites (DAMs), 296 up-regulated and 237 down-regulated DAMs, and 426 up-regulated and 237 down-regulated DAMs, were identified in 3Y, 5Y and fruits respectively (Table S4). These DAMs were divided into 10 categories by unit variance scaling treatment according to the relative abundance (Fig. 3A and B; Table S4). In subclass 1 and 2, a total of 132 DAMs showed a continuously decreasing trend in relative abundance with the rhizomes’ growth years, of which the number of steroids was the largest, accounting for 25%. Contrarily, in subclass 4, 7 and 10, the relative abundance of 308 DAMs increased with the rhizomes’ growth year, of which the number of flavonoids, alkaloids and amino acids and derivatives was larger. In subclass 5 and 6, a total of 416 DAMs’ relative abundance in fruits were higher than rhizomes, especially in subclass 6, which suggested that these DAMs might be the special contents in fruits; Among them, the number of flavonoids, lipids and phenolic acids was bigger (Fig. 3A and B; Table S4). Moreover, the FC value of DAMs showed that the common top ten up-regulated DAMs in 1Y vs. 3Y and 1Y vs. 5Y were Menatetrenone (Vitamin K2), 5-Hydroxy-L-tryptophan and N-benzoyl-2-aminoethyl-β-D-glucopyranoside, and the common top ten down-regulated DAMs in 1Y vs. 3Y and 1Y vs. 5Y were 6-O-Acetylarbutin and 4-Acetamidobutyric acid (Fig. 3C and D). The top ten up-regulated and down-regulated DAMs in 3Y vs. 5Y were mainly flavonoid compounds and nucleotides and derivatives compounds, respectively (Fig. 3E). The top ten up- and down-regulated DAMs in 1Y vs. F were primary flavonoid compounds (Fig. 3F). What’s more, the 8 of 22 flavonoids identified by Han et al. [20] showed significant different content in 1Y, 3Y and 5Y rhizomes according to our metabolomic results, 13 flavonoids were not identified in our results, and only one flavonoid showed a similar content among different rhizomes (Table S5).

### Comparative transcriptomic analysis in fruits and different year rhizomes of *P. cyrtonema*

**Fig. 4:**
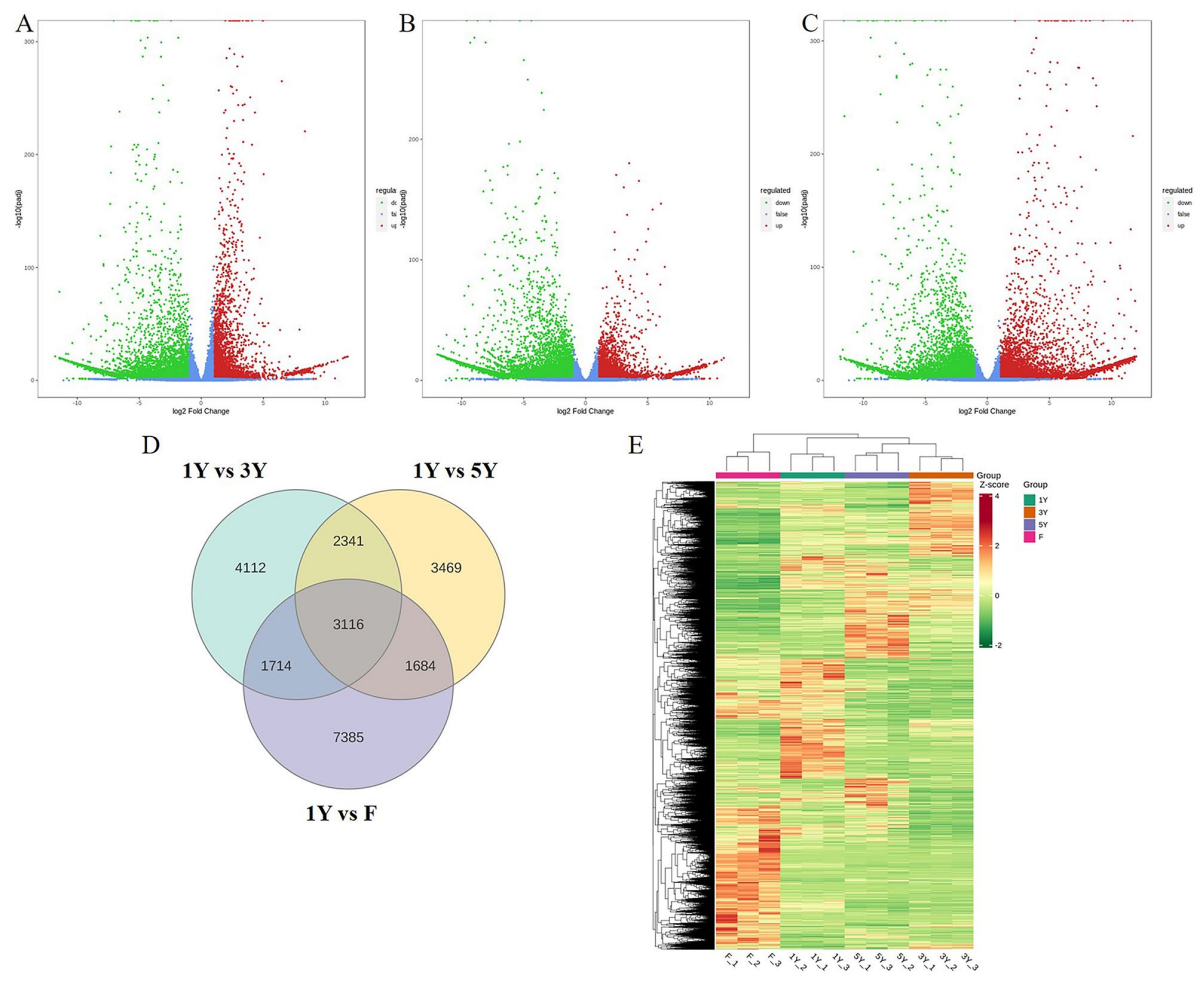
Analysis of DEGs in fruits and different year-old rhizomes of *P. cyrtonema*. Volcano plot of the DEGs in 1Y vs. 3Y **(A)**, 1Y vs. 5Y **(B)** and 1Y vs. F **(C)**. **(D)** Venn diagram of the DEGs. **(E)** Expression of DEGs in fruits and different year-old rhizomes

Three biological replicates of RNA-seq in different samples was profiled and good repetition among the three biological replicates in each sample were obtained (Fig. S2), which produced 151.0, 166.0, 156.6 and 160.0 million raw reads in 1Y, 3Y, 5Y and F, respectively (Table S6). The final assemblies were well annotated and made available, for the transcript N50 values was 1 434 bp and unigene N50 values was 1 628 bp, and the total BUSCO score was 97.3% (Table S6). The differential gene expression analysis was performed, and a total of 3 892, 3 796, and 6 533 up-regulated differentially expressed genes (DEGs) and 7 391, 6 814 and 7 366 down-regulated DEGs (*P* < 0.05) were identified in 1Y vs. 3Y, 1Y vs. 5Y and 1Y vs. F, respectively (Fig. 4A and C, Table S7). There were 5 457 DEGs shared between 1Y vs. 3Y and 1Y vs. 5Y, their expressions in 1Y were significant different with 3Y or 5Y. 7 385 DEGs in 1Y vs. F might be the genes that specifically differentially expressed in fruits (Fig. 4D). The HCA of all DEGs was performed to show the global gene expression pattern at different samples (Fig. 4E).

Furthermore, 12 DEGs related to polysaccharide, flavonoid and terpenoid biosynthesis were selected for qRT-PCR validation (Fig. S3), for example, *Cluster-50324.5* (*Fructokinase*, *FRK*), *Cluster-60494.5* (*chalcone synthase*, *CHS*) and *Cluster-66805.27* (*squalene synthase*, *SQS*). Most of the expression patterns of these selected DEGs were basically consistent with the RNA-seq data, which indicated the RNA-seq data were reliable.

### Correlation analysis between DAMs and DEGs

To illustrate the relationships between DAMs and DEGs, the nine-quadrant maps were drawn (Fig. S4). The results showed that, most DAMs (835 in 1Y vs. 3Y, 796 in 1Y vs. 5Y, and 859 in 1Y vs. F) and DEGs (28 775 in 1Y vs. 3Y, 29 174 in 1Y vs. 5Y, and 30 484 in 1Y vs. F, Table S8) were in quadrant 2, 4, 6, and 8, indicating no correspondence between these metabolites and genes. However, the DAMs and DEGs in quadrants 3 and 7 were positively correlated. KEGG pathway analysis of these DAMs and DEGs in quadrants 3 and 7 were conducted (*P* < 0.05), and the flavonoid biosynthesis pathway was identified in both 1Y vs. 3Y and 1Y vs. F (Fig. S4D, 4E). The DAMs and DEGs in quadrants 3 and 7 were further analyzed as follows.

### DEGs and DAMs involved in polysaccharides biosynthesis

According to the previous studies [8, 17], fifteen ‘Carbohydrate metabolic’ subgroups, which were involved in polysaccharides biosynthesis, were further analyzed to enhance our comprehension of polysaccharide biosynthesis in *P. cyrtonema*. It’s interesting that, the down-regulated DEGs (or DAMs) in quadrants 7 were more than the up-regulated DEGs (or DAMs) in quadrants 3 of the same pathways, in both 1Y vs. 3Y and 1Y vs. 5Y (Table S9). What’s more, DEGs in quadrants 7 between 1Y vs. 3Y and 1Y vs 5Y, which were enriched in ‘Starch and sucrose metabolism’ (ko00500) and ‘Amino and nucleotide sugar metabolism’ (ko00520) pathways, were further annotated. A total of 112 DEGs with higher expression in 1Y rhizome and encoding key enzymes were selected, including *beta-fructofuranosidase* (*sacA*), *mannose-1-phosphate guanylyltransferase* (*GMPP*), *phosphomannomutase* (*PMM*) et al. (Table S10). Furthermore, in 1Y vs. F, there were 390 DEGs identified in ‘Starch and sucrose metabolism’ (ko00500) and ‘Amino and nucleotide sugar metabolism’ (ko00520) pathways, and the number of DAMs in quadrants 3 were bigger than these in quadrants 7 (Table S10).

### DEGs and DAMs involved in flavonoids biosynthesis

‘Flavonoid biosynthesis’ (ko00941) was enriched by the DEGs and DAMs in quadrants 3 and quadrants 7. For 1Y vs 3Y, only one DAMs (Epiafzelechin) and twenty-one DEGs were up-regulated in ko00941. The down-regulated DAMs were Naringenin-7-O-Neohesperidoside (Naringin), Pinocembrin (Dihydrochrysin), Homoeriodictyol and so on (Table S11). Genes encoding *trans-cinnamate 4-monooxygenase*, *5-O-(4-coumaroyl)-D-quinate 3’-monooxygenase*, *caffeoyl-CoA O-methyltransferase* (*CCoAOMT*, including *Cluster-50095.0*, *Cluster-50095.1*, *Cluster-50095.2* and *Cluster-50095.3*), *chalcone isomerase* (*CHI*, including *Cluster-43708.0*, *Cluster-51631.0* and *Cluster-51631.1*) and *anthocyanidin reductase* (*ANR*) were up-regulated (Table S12). Whereas *naringenin 3-dioxygenase*, *CCoAOMT* (*Cluster-69209.0*, *Cluster-69209.1*), *chalcone synthase* (*CHS*), *CHI* (*Cluster-39698.0*, *Cluster-51631.3* and *Cluster-63757.0*), *anthocyanidin synthase* (*ANS*), *flavonol synthase* (*FLS*), *flavonoid 3’-monooxygenase* (*F3’H*), *shikimate O-hydroxycinnamoyltransferase* (*HCT*), *bifunctional dihydroflavonol 4-reductase/flavanone 4-reductase* and *phlorizin synthase* were down-regulated (Table S12). For 1Y vs 5Y, forty-one DEGs were identified in ko00941 (Table S12). Among these DEGs, *ANS* (*Cluster-62399.5*, with log2 FC = 1.07) and *HCT* were up-regulated, while *CCoAOMT*, *CHS*, *CHI* and *ANS* (*Cluster-46008.0* and *Cluster-46008.2*, with log2 FC = -5.81 and − 8.30, respectively) were down-regulated. The down-regulated DAMs were Pinocembrin (Dihydrochrysin), Homoeriodictyol and so on, and most of these down-regulated DAMs exhibited high log2 value (Table S11). For 1Y vs F, six DAMs and seventeen DEGs were up-regulated in ko00941 (Table S11, 12). Among these DAMs, Gallocatechin and Catechin were up-regulated with the log2 FC over 5, Pinocembrin (Dihydrochrysin), Kaempferol (3,5,7,4’-Tetrahydroxyflavone), Quercetin and 3,4,2’,4’,6’-Pentahydroxychalcone were down-regulated with the log2 FC less than − 5. At the meanwhile, *CHI*, *ANR*,* phlorizin synthase*, *CCoAOMT*, *CHS*, *HCT* and so on were differentially expressed with a high FC (Table S11, S12).

### DEGs and DAMs involved in triterpene saponins biosynthesis

To illustrate triterpene saponins biosynthesis in different year rhizomes and fruits, ‘Terpenoid backbone biosynthesis’ (ko00900) and ‘Carotenoid biosynthesis’ (ko00906) pathways were further analyzed by the DEGs and DAMs in quadrants 3 and quadrants 7. Among these DEGs, *1-deoxy-D-xylulose-5-phosphate synthase* (*DXS*), *geranylgeranyl diphosphate synthase* (*GGPS*) and *zeaxanthin epoxidase* (*ZEP*) were identified (Table S13). However, very few DAMs in quadrants 3 and quadrants 7 were identified. Mevalonic acid (MVA) and Abscisic acid (ABA) were identified in quadrants 3 of 1Y vs F, and only ABA were identified in quadrants 7 of both 1Y vs 3Y and 1Y vs 5Y (Table S11).

### DEGs and DAMs involved in alkaloids biosynthesis

For alkaloids biosynthesis, only ‘Biosynthesis of various alkaloids’ (ko00996) was enriched according to the DEGs and DAMs in quadrants 3 and quadrants 7. Among the DAMs, when compared with the 1Y rhizomes, L-Tryptophan was up-regulated in both 3Y and 5Y rhizomes, while down-regulated in fruits. Shikimic acid and phosphoenolpyruvate were significantly down-regulated in 5Y rhizomes, both with the log2 FC less than − 13 (Table S11). What’s more, Anthranilic acid was down-regulated in fruits. It’s interesting that, a *cholesterol 22-hydroxylase* was identified among the DEGs, which was similar with the *cytochrome P450 CYP72A219-like (A)* (Table S13).

### Co-expression network analysis

**Fig. 5:**
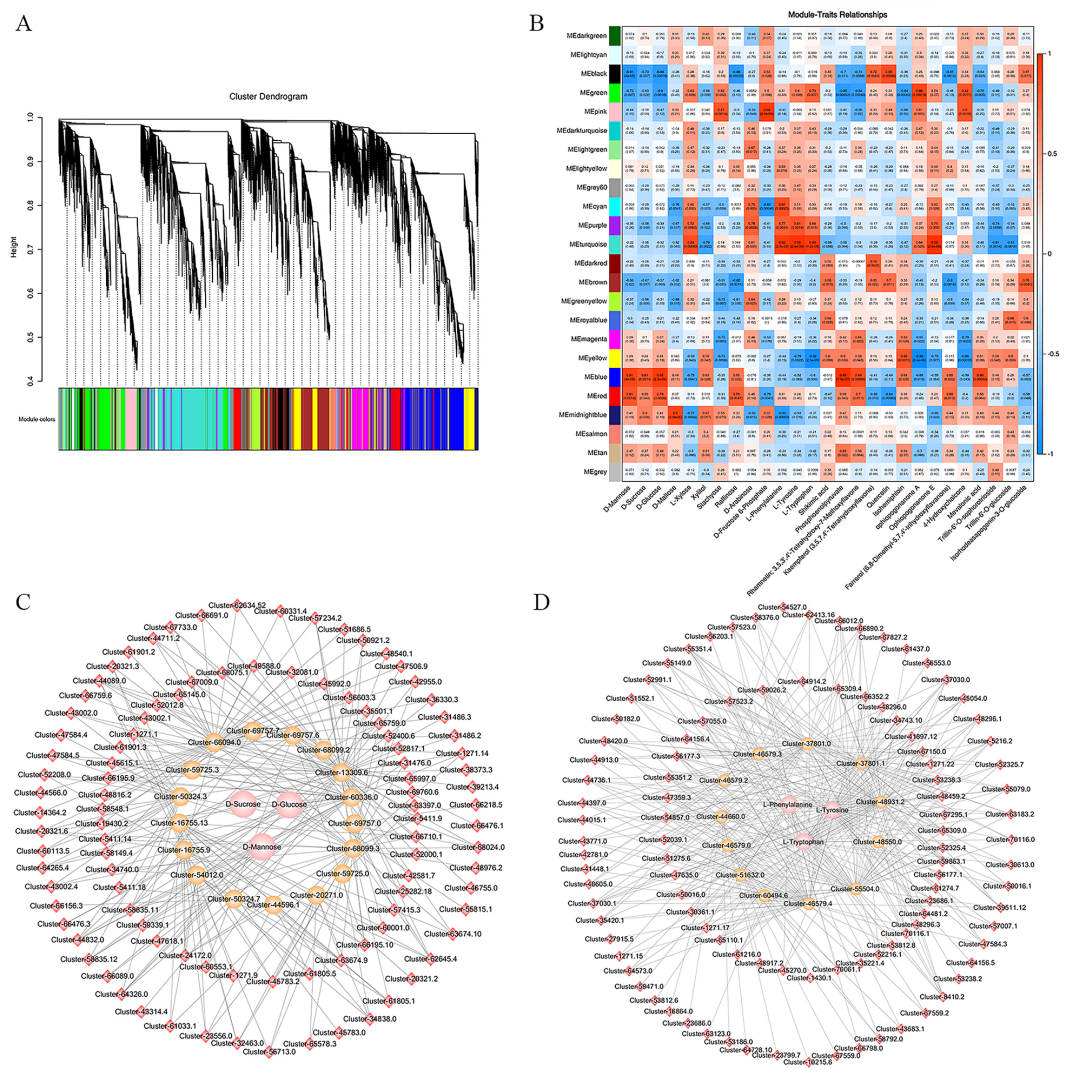
Metabolic and transcriptomic correlation analysis in fruits and different years rhizomes of *P. cyrtonema*. **(A)** Dendrogram showing co-expression modules identified by WGCNA in fruits and different years rhizomes. The major tree branches constitute 24 modules labeled with different colors. **(B)** Heat map of the module-metabolite correlations. Each row corresponds to a module indicated by different colors. Each column corresponds to a metabolite. Blue color represents a negative correlation. Red color represents a positive correlation. **(C)** The regulatory network of three solubar sugars in *P. cyrtonema*. The pink circles represent soluble sugars. Yellow circles represent structural genes relate to soluble sugars metabolism in fruit and different year rhizomes. Red diamond represents TFs identified in the same module whose transcripts are correlated with the expression of structural genes. **(D)** The regulatory network of three aromatic amino acids in *P. cyrtonema*. The pink circles represent aromatic amino acids. Yellow circles represent structural genes relate to aromatic amino acids metabolism in fruit and different year rhizomes. Red diamond represents TFs identified in the same module whose transcripts are correlated with the expression of structural genes

To gain further insight into the gene regulatory network and identify potential key regulatory factors related to polysaccharides, flavonoids, saponins and alkaloids biosynthesis and accumulation, the Weighted gene-co-expression network analysis (WGCNA) was conducted to investigate co-expression networks of the DEGs. A total of 24 co-expression modules were identified according to the similar expression patterns (Fig. 5A; Table S14). The heatmap of module-trait correlations showed the accumulation of transcripts in blue module was mainly correlated with polysaccharides biosynthesis associated metabolites, such as D-Mannose, D-Sucrose and D-Glucose (Fig. 5B). In addition, the accumulation of transcripts for the turquoise module was highly correlated with the aromatic amino acids, including L-Phenylalanine, L-Tyrosine and L-Tryptophan (Fig. 5B).

To generate the potential regulatory network associated with sugar metabolism, the structural genes in the blue module were further analyzed (absolute value of Pearson correlation coeffcient > 0.9, p-value < 0.01) (Fig. 5A; Table S14). We identified 17 polysaccharides biosynthesis related structural genes, including two *beta-fructofuranosidase* (*sacA*), five *fructokinase* (*scrK*), three *mannose-6-phosphate isomerase* (*MPI*), one *phosphomannomutase* (*PMM*), four *mannose-1-phosphate guanylyltransferase* (*GMPP*), one *glucose-6-phosphate isomerase* (*GPI*) and one *3*,*5-epimerase/4-reductase* (*UER1*) genes in the blue module (Table S14) with high correlation with the accumulation of D-Mannose, D-Sucrose and D-Glucose. By correlating the accumulation patterns of transcripts and the potential binding affinity of the structural genes associated with polysaccharides biosynthesis, we identified 100 transcription factors (TFs) including *C2C2*, *MYB* and *bZIP*, which expression were highly correlated with the 17 polysaccharides biosynthesis related structural genes in blue module and constructed a correlation network (Fig. 5C; Table S15).

Aromatic amino acids including Phenylalanine, tyrosine and tryptophan, which can be further transformed to kinds of flavonoids or alkaloids. To generate the potential regulatory network associated with aromatic amino acids metabolism, the structural genes in turquoise module were further analyzed (absolute value of Pearson correlation coeffcient > 0.9, p-value < 0.01) (Fig. 5A; Table S14). We identified 12 aromatic amino acids metabolism related structural genes, including one *CHS*, four *CHI*, one *flavanone 3-hydroxylase* (*F3H*), one *cinnamoyl-CoA reductase* (*CCR*), one *cinnamyl-alcohol dehydrogenase* (*CAD*), one cholesterol 22-hydroxylase and three shikimate O-hydroxycinnamoyltransferase (*HCT*) in turquoise module (Fig. 5D; Table S16) with high correlation with the accumulation of L-Phenylalanine, L-Tyrosine and L-Tryptophan. Additionally, 102 TFs including *C2H2*, *GRAS* and *NAC* were identified with highly correlation with the 12 aromatic amino acids metabolism related structural genes in turquoise module and constructed a correlation network (Fig. 5D; Table S16).

## Discussion

Recently, the UHPLC-Q-TOF-MS untargeted metabolomic analysis had identified 1126 metabolites in *P. cyrtonema* and *P. sibirucum* [19]; the widely-targeted metabolome analysis in 15 genotypes of *P. cyrtonema* had identified 22 flavonoids [20]. However, to date, there is a lack of research comparing the metabolites profiles in different year-old node of rhizomes. What’s more, the fruits of *P. cyrtonema* also had edible or medical value according to our Chinese ancient books [1], while there is no related report about the metabolites in fruits up to now. Here, we conducted the UPLC-MS/MS-based widely targeted metabolomics and transcriptomic analyses in fruits and 1Y, 3Y and 5Y rhizomes, to determine the metabolites profile and key regulatory factors related to these metabolites.

In the present study, 1338 metabolites were detected and quantified from fruits and 1Y, 3Y, 5Y rhizomes of *P. cyrtonema*, which were further classified into 13 categories according to the function or structure similarity. It’s interesting that, the relative content of flavonoids, lignans and coumarins, phenolic acids and alkaloids increased with the rhizomes’ growth year (Fig. 2B). While the relative content of steroids and lipids decreased with the rhizomes’ growth year. Moreover, the total polysaccharide content in rhizomes also increased with the growth year at first, and the 3Y and 4Y rhizomes had the higher value, while decreased in 5Y rhizomes (Fig. 1D). Besides polysaccharides and saponins, flavonoids and phenolics in rhizomes also have unique therapeutic role in prevention and treatment of diabetes [21]. The wogonin in *P. cyrtonema* rhizomes exhibited the activity of against HepG-2 cell lines [3]. Huang et al [22] isolated 3-(2’-furaldehyde-5’-methoxymethyl)5,6,7,8-tetrahydroindolizin-8-one (an alkaloid) from *P. cyrtonema* showed the protective effect against Aβ_25−35_ induced oxidative stress damage. Hence, when *P. cyrtonema* rhizomes were used for medicinal purpose, the growth year should be standardization, for the content of each compound in different year rhizomes were various. For example, the 8 of 22 flavonoids identified by Han et al [20] showed different content in 1Y, 3Y and 5Y rhizomes according to our metabolomic results (Table S5). Hence, this study could provide a useful reference for setting the related standardization.

Up to now, fruits in *P. cyrtonema* were usually used to get seeds for propagation, and there is no report about their utilization in food or medicine. In this study, fruits were distinguished from rhizomes by PC1 and PC2 (Fig. S1A), which indicated the metabolites in fruits were significantly different with those in rhizomes. Moreover, the relative content of nucleotides and derivatives, flavonoids, organic acids, steroids and lipids in fruits were higher than rhizomes (Fig. 2B), and the total polysaccharides content in fruits was 74.4 mg/g, which was equal with 1Y and 5Y (Fig. 1D). Therefore, the medicinal and edible value of fruits might be different with rhizomes.

In this study, we combined metabolome and transcriptome analyses to explore the dynamic accumulation and regulatory network of metabolites in *P. cyrtonema* rhizomes. For polysaccharides biosynthesis, some key enzymes genes were selected, including *sacA*, *GMPP* and *PMM*, which had higher expression in 1Y (Table S10). In previous research, *sacA* has been verified relating to biosynthesis of plant polysaccharides [23]; overexpression of *GMPP* in *Trichoderma reesei* led to a two-fold increase in GDP-mannose [24]; overexpression of *PMM* could increase the bioactivities and production of Ganoderma exopolysaccharides [25]. However, the polysaccharides content in 1Y was lower than 3Y, which suggested the polysaccharides might be primarily synthesized in 1Y and could be transferred to other rhizomes. The 5Y rhizomes might be too far from the 1Y rhizomes to get more polysaccharides, and the polysaccharides could be consumed at the same time, so the polysaccharides in 5Y rhizomes were lower. According to the WGCNA analysis, 100 TFs including bZIP, ERF, C2C2, C3H, MYB and MADS, which expression were highly correlated with the 17 polysaccharides biosynthesis related structural genes in blue module (Fig. 5C; Table S15). These TFs with higher expression in 1Y should be the putative regulators controlling the sugar metabolism in *P. cyrtonema* rhizomes.

In flavonoids biosynthesis, when compared with 1Y, *CCoAOMT* (*Cluster-69209.0*, *Cluster-69209.1*), *CHI* (*Cluster-39698.0* and *Cluster-63757.0*), *FLS* (*Cluster-53025.2* and *Cluster-53025.4*), *F3’H* (*Cluster-36454.0* and *Cluster-46704.0*) and *ANS* (*Cluster-46008.0* and *Cluster-46008.2*) were significantly down-regulated in both 3Y and 5Y (Table S11, S12). *ANS*, *FLS*, *CCoAOMT* and *CHI* were very important in the biosynthesis of plant flavonoids [26–28], and overexpression of *PcCHI*, *PcCHS1*, *2* showed the content of 12 flavonoids were significantly changed in tobacco leaves [20]. In our results, these genes had higher expression in 1Y, which might indicate flavonoids biosynthesis also mainly take place in 1Y, though few copies of these key enzymes genes had up-regulated in 3Y or 5Y (with low log2 FC). The old rhizomes might accumulate more flavonoids as the time went on, which was consisted with the metabolic results (Fig. 2B).

For triterpene saponins biosynthesis, DEGs and DAMs in Ko00900 and Ko00906 were analyzed according to the previous study [8]. The expression trend of *DXS*, *GGPS* and *ZEP* were consistent with the relative content variation trend of their catalytic reaction products (Table S11). *DXS* was the first step and the key regulatory enzyme of the methylerythritol-4-phosphate (MEP) pathway in plants [29]. The MEP pathway was the main pathway to synthesize the isoprenoids, which could be further transformed to all kinds of terpenoids. In plants, GGPP (geranylgeranyl diphosphate) was catalyzed by *GGPS* and mediated the function of various metabolites [30]. *ZEP* could epoxidate the xanthophyll zeaxanthin to violaxanthin and involved in the light-dependent growth of the marine alga *Nannochloropsis oceanica* [31]. As the Fig. 2B showed that, the relative content of terpenoids in fruits and each node of rhizomes did not show significant difference, which indicated that the terpenoids might also mainly synthesize in 1Y rhizomes and fruit, but not accumulate year by year.

For alkaloids biosynthesis, DEGs and DAMs in ko00996, ko01063, ko01064, ko01065 and ko01066 were analyzed. The expression of *cytochrome P450 CYP72A219-like (A)* was identified consistent with alkaloids biosynthesis (Table S11). In previous study, *CYP72A219* highly expressed in the saffron pistils and was the candidate gene involved crocin biosynthesis [32]; In *Amaranthus palmeri*, *CYP72A219* highly consistently expressed in the all treated tolerant biotypes and was the candidate gene for contributing tolerance to glufosinate herbicide [33]. Hence, the *cytochrome P450 CYP72A219-like (A)* identified in this study might be the key genes in alkaloids biosynthesis in *P. cyrtonema*.

The accumulation of plant secondary metabolites is affected by TFs, which integrate internal and external signals to regulate the expression of enzyme genes [34]. In the present study, according to the WGCNA result, we identified 100 TFs, which expression were highly correlated with the polysaccharides biosynthesis related structural genes (Table S15). Among these TFs, the top three families were *C2C2*, *MYB* and *bZIP*. Moreover, 102 TFs were identified with highly correlation with the aromatic amino acid metabolism related structural genes (Table S16). Among these TFs, the top three families were *C2H2*, *GRAS* and *NAC*. In *Artemisia annua*, the overexpression of *AaNAC1* can enhance the content of artemisinin and increase the tolerance to botrytis cinerea and drought [35]. *OpNAC1* can improve the biosynthesis of camptothecin through targeting loganic acid O-methyltransferase in *Ophiorrhiza pumila* [36]. Hence, the TFs identified in this study might be the potential regulators that regulating the sugars and aromatic amino acids metabolism in *P. cyrtonema* rhizomes and fruits.

## Conclusion

**Fig. 6:**
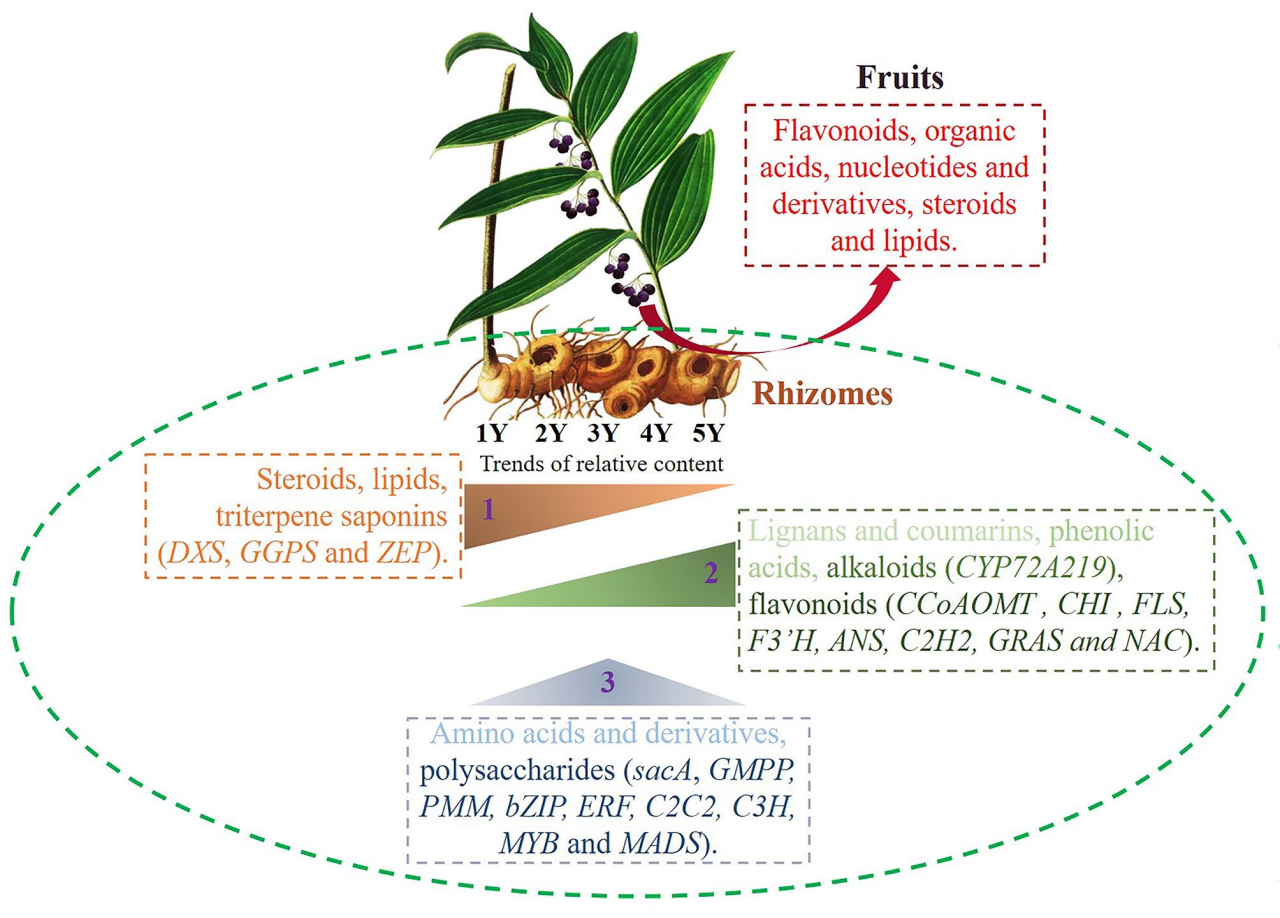
The regulatory and accumulation patterns in different year rhizomes and fruits in *P. cyrtonema*. Trends 1, 2 and 3 represents the relative content decreased with the growth year, increased with the growth year, and the higher relative content occurred in 3Y, respectively. The metabolites belonged to the related trends were showed in the lateral boxes, and the feature genes involved in the metabolites was showed in the brackets. The metabolites with higher relative content in fruits were showed in the red box

In this study, we performed the integrative analyses of metabolome and transcriptome, to reveal the dynamic accumulation and regulatory network in different year-old node of rhizomes and fruits of *P. cyrtonema*. We found the total polysaccharides content was higher in 3Y and 4Y rhizomes. In addition, the relative content of phenolic acids, flavonoids, lignans and coumarins and alkaloids increased with the rhizomes’ growth year, the relative content of steroids and lipids decreased with the rhizomes’ growth year, and the relative content of nucleotides and derivatives, flavonoids, organic acids, steroids and lipids were higher in fruits. What’s more, the regulatory network in polysaccharides, flavonoids, triterpene saponins and alkaloids biosynthesis were further analyzed, and key genes in sugar and aromatic amino acids metabolites were constructed to further illustrate the biosynthesis of polysaccharides, flavonoids and alkaloids (Fig. 6). Interestingly, according to our results, the polysaccharides and flavonoids might primarily synthesize in 1Y and could be transferred to other rhizomes. While the terpenoids might also mainly synthesize in 1Y rhizomes and fruit, but not accumulate year by year. This study can deepen our understanding of the molecular mechanism of the main compounds’ biosynthesis and accumulation patterns, and provide the reference theory for standardize production and potential candidate genes for molecular breeding in *P. cyrtonema*.

## Materials and methods

### Plant materials

In September 2022, *P. cyrtonema* were collected from Forest Farm of Xuefeng mountain (27°12′N, 110°21′E) at an elevation of 600 m, Huaihua, Hunan Province, China. These plants were cultivated in imitating wild condition by Hunan Xinhui pharmaceutical Co. Ltd. More than sixty 5-year-old plants with great growth consistency were randomly selected, and over twenty plants were mixed as one sample. The roots, stems, leaves, fruits and different year-old node of rhizomes were cleaned by pure water and dried on filter papers. Samples for metabonomic and transcriptomic analysis were frozen in liquid nitrogen immediately and then stored at -80 °C. Samples for total polysaccharides content analysis were dried to a constant weight at 60 ℃.

### Total polysaccharides content analysis

Total polysaccharides were extracted and tested from the dried *P. cyrtonema* samples according to the method described in Chinese Pharmacopoeia. Over three repetitions have been conducted in each sample and the statistical analysis has been performed by SPSS 22.0 software.

## Widely targeted metabolomics analysis

The 1-year-old (1Y), 3-year-old (3Y), 5-year-old (5Y) rhizomes and fruits (F) of *P. cyrtonema* were chosen for widely targeted metabolomics analysis, and each group had three biological replicates. This part of experiments was conducted by MetWare Biotechnology Co., Ltd. (Wuhan, China).

All samples were vacuum-freeze dried using a lyophilizer (Scientz-100 F) and then ground to powder at 30 Hz in a grinder (MM 400, Retsch, Germany) separately. 50 mg powder was dissolved with 70% methanol (1.2 mL), and vortexed 30 s per 30 min for 6 times totally. Then centrifugation for 3 min at 12 000 rpm, the supernatant was transferred to 0.22 μm filters, and stored in the sample bottles.

UPLC (ExionLC™ AD) and MS/MS (Applied Biosystems 6500 QTRAP) were used for UPLC-MS/MS analysis. Chromatographic separation was conducted on an Agilent SB-C18 column (1.8 μm, 2.1 mm*100 mm) using 0.1% methanoic acid in ultrapure water and 0.1% methanoic acid in acetonitrile as mobile phase A and B, respectively. The elution program was followed as: 95:5 V(A)/V(B) at 0 min, 5:95 V(A)/V(B) at 9 min, remained unchanged to the 10th min, 95:5 V(A)/V(B) at 11.1 min, and remained unchanged to the 14th min. The column temperature was 40 ℃, the flow rate was 0.35 mL/min, and the injection volume was 2 µL. The relative abundance of the compounds was represented by each peak area.

Mass data acquisition was conducted in ion spray voltage of positive (5.5 kV)/negative (-4.5 kV) mode, with electrospray ionization temperature of 550 ℃. Ion source gas I, gas II and curtain gas were set as 50, 60 and 25 psi respectively. The multiple reaction monitoring (MRM) mode was used for triple quadrupole (QQQ) scanning. The individual MRM transitions were conducted with specific Collision energy (CE) and declustering potential (DP) optimization. Based on the eluted metabolites within every period, a specific MRM ion pair would be monitored.

The metabolites were verified by comparing the m/z values, the fragmentation patterns and the retention time (RT) with the standards in a database constructed by MetWare. Quantification of the metabolites was completed by using the MRM analysis of the QQQ mass spectrometry. The targets’ precursor ions were selected by quadrupole, and the ions related to other molecular weight compounds would be removed. The precursor ions were broken into several fragments, and the characteristic fragment in demand would be selected through the QQQ. Then the metabolite’s relative abundance was determined according to the signal strength of the characteristic fragment. When obtaining compound profiles for all samples, the MS peak areas of different metabolites would be integrated. Then MultiQuant software was applied to process the MS peak and correct the same compound’s MS peaks in different samples. The relative abundance of the compounds was represented by each peak area [19, 37]. Principal component analysis (PCA) on different samples for comparison was conducted by ‘prcomp’ in R software (www.r-project.org/). The DAMs were determined by log2 fold change (FC) ≥ 2 or ≤ 0.5 and variable importance in projection (VIP) ≥ 1.

### Transcriptomic analysis

The TRIzol RNA extracting kit (ThermoFisher SCIENTIFIC) was used to extract the total RNA, the Qubit 2.0 fluorimeter was used to measure the RNA concentration, and the RNA integrity was detected by the Agilent 2100 Bioanalyzer (Agilent Technologies, CA, USA). cDNA library was obtained by the PCR enrichment and the Illumina Novaseq6000 system platform was used to sequence. The CASAVA base recognition was performed to convert the image data which was obtained via high-throughput sequencer, into a large amount of high-quality raw data. Then the fastq software [38] was used to filter the sequencing data to get clean reads. After that, the Trinity software was used to stitch the clean reads together [39]. The overall quality of the transcriptome was assessed for completeness, using Benchmarking Universal Single-Copy Orthologs tool (BUSCO version 5.4.3) [40]. Then Fragments Per Kilobase of transcript per million fragments mapped (FPKM) was applied to measure the gene expression levels [41]. DESeq2 software was used to identify the DEGs under p-value < 0.05 and |log2FC| ≥ 1.

### qRT-PCR

For DEGs, total RNA was reverse-transcribed by using ReverTra Ace^®^ qPCR-RT Master Mix with gDNA Remover (TOYOBO) according to the manufacturer’s protocol and the *elongation factor 1-α* (*EF1α*) was used as an internal reference [8]. Specific primers of the target genes were designed by primer 5.0 software. qRT-PCR was performed by Applied Biosystems QuantStudio 5 with Hieff^®^ qPCR SYBR Green Master Mix (Low Rox Plus) (YEASEN) according to the manufacturer’s protocol. The relatively expression of target genes were calculated by 2^−ΔΔCt^ method [42]. All primer sequences were listed in Table S1.

### Weighted gene-co-expression network analysis (WGCNA)

DEGs identified by the transcriptomic analysis were used to obtain the co-expression network modules by WGCNA package in R. The co-expression modules were generated via automatic network construction function with the default parameters, mergeCutHeight was 0.25, powerEstimate was 18 and minModuleSize was 50. The eigengene values were calculated in each module, which was applied to search the association with the main compounds in *P. cyrtonema*. The module member-ship (MM) value was obtained by using the signedKME function of the WGCNA package to analyze the correlation between gene expression and module eigengene. These networks were visualized by Cytoscape v.3.7.2 Software.

### Electronic supplementary material

Below is the link to the electronic supplementary material.

Supplementary Material 1

Supplementary Material 2

Supplementary Material 3

Supplementary Material 4

Supplementary Material 5

Supplementary Material 6

Supplementary Material 7

Supplementary Material 8

Supplementary Material 9

Supplementary Material 10

Supplementary Material 11

Supplementary Material 12

Supplementary Material 13

Supplementary Material 14

Supplementary Material 15

Supplementary Material 16

Supplementary Material 17

Supplementary Material 18

Supplementary Material 19

Supplementary Material 20

Supplementary Material 21

## Data Availability

The obtained raw reads of RNA-seq were deposited in SRA (NCBI Sequence Read Archive) database under the accession number of PRJNA948546 (https://dataview.ncbi.nlm.nih.gov/object/PRJNA948546?reviewer=omcljvf8mcsatgsgs2i9c5tnud).

## Declarations

### Plant specimen

All the plants were identified as *Polygonatum cyrtonema* Hua by associate Professor Yeman Liu (Hunan University of Chinese Medicine). The authenticated specimens (Vouch ID: 20220903) are kept in the TCM and Ethnomedicine Innovation & Development International Laboratory, Innovative Material Medical Research Institute, School of Pharmacy, Hunan University of Chinese Medicine. All local, national or international guidelines and legislation were adhered to in the production of this study.
