# Supplementary material for: Integrative analyses of metabolome and transcriptome reveal the dynamic accumulation and regulatory network in rhizomes and fruits of Polygonatum cyrtonema Hua
Source: BMC Genomics. 2024 Jul 19;25:706. doi: 10.1186/s12864-024-10608-4 (PMC11264994; doi:10.1186/s12864-024-10608-4)
Supplement: Supplementary file 1 — Supplementary Material 1 [file 12864_2024_10608_MOESM1_ESM.docx]

**Legends for Supplementary materials**

**Fig. S1** Metabolite accumulation in fruits and different year-old rhizomes of *P. cyrtonema*. **(A)** PCA of metabolites in different groups. **(B)** HCA of metabolites in different groups. **(C)** Pearson’s Correlation Coeffcients of the metabolite expression profile.

**Fig. S2** Pearson’s Correlation Coeffcients of the gene expression profile.

**Fig. S3** Validation for DEGs by qRT-PCR. A result from RNA-seq. B result from qRT-PCR. The numbers are log_2_^X^-normalized ratio values. Red color corresponds to higher gene expression levels when compared with 1Y. Green color represents lower gene expression levels.

**Fig. S4** Integrated analysis of DAMs and DEGs. **(A)** Nine-quadrant map of DAMs and DEGs. **(B)** KEGG pathway analysis of DAMs and DEGs in quadrants 3 and 7.

**Table S1** All Primers used in qRT-PCR.

**Table S2** All metabolites identified in fruits and rhizomes of *P. cyrtonema*.

**Table S3** The raw data for Fig. 2B.

**Table S4** DAMs in different groups.

**Table S5** Results of the 22 reported ﬂavonoids compounds in our metabolome.

**Table S6** Summary of RNA-seq data.

**Table S7** FPKM values of DEGs in different samples.

**Table S8** FPKM values of DEGs in quadrant 2, 4, 6, and 8 of the correlation analysis between DAMs and DEGs

**Table S9** Number of DEGs and DAMs involved in polysaccharides biosynthesis.

**Table S10** The polysaccharide biosynthesis related enzyme genes in quadrants 7.

**Table S11** DAMs involved in flavonoids, triterpene saponins and alkaloids biosynthesis in quadrants 3 and 7.

**Table S12** DEGs involved in flavonoids biosynthesis in quadrants 3 and 7.

**Table S13** DEGs involved in triterpene saponins and alkaloids biosynthesis in quadrants 3 and quadrants 7.

**Table S14** Summary of co-expression unigene modules.

**Table S15** The regulatory network of sugars metabolism in *P. cyrtonema*.

**Table S16** The regulatory network of aromatic amino acids metabolism in *P. cyrtonema*.
