# Supplementary figures and images for: Integrative analyses of metabolome and transcriptome reveal the dynamic accumulation and regulatory network in rhizomes and fruits of Polygonatum cyrtonema Hua
Source: BMC Genomics. 2024 Jul 19;25:706. doi: 10.1186/s12864-024-10608-4 (PMC11264994; doi:10.1186/s12864-024-10608-4)

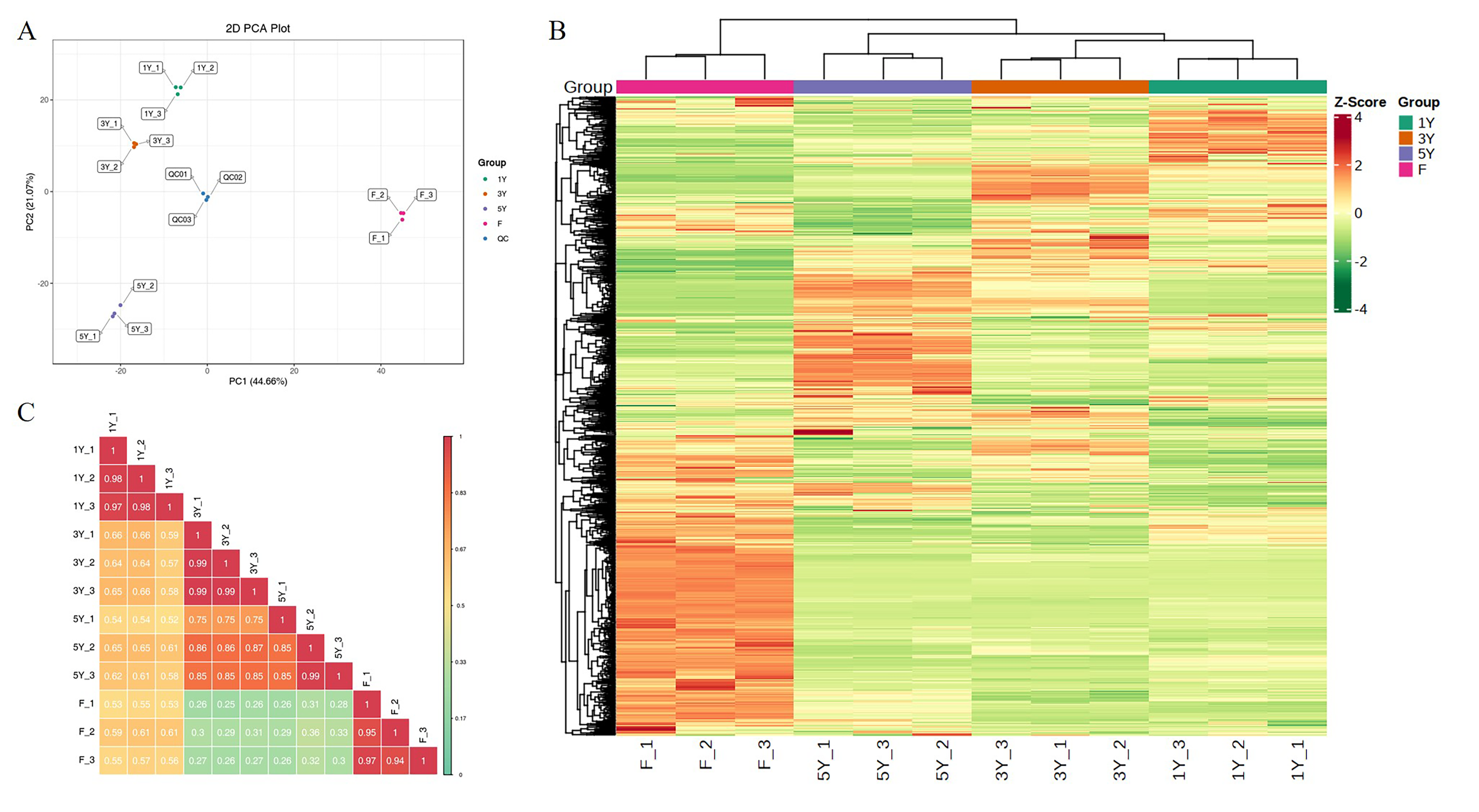

Supplement: Supplementary file 2 — Supplementary Material 2 [file 12864_2024_10608_MOESM2_ESM.png]

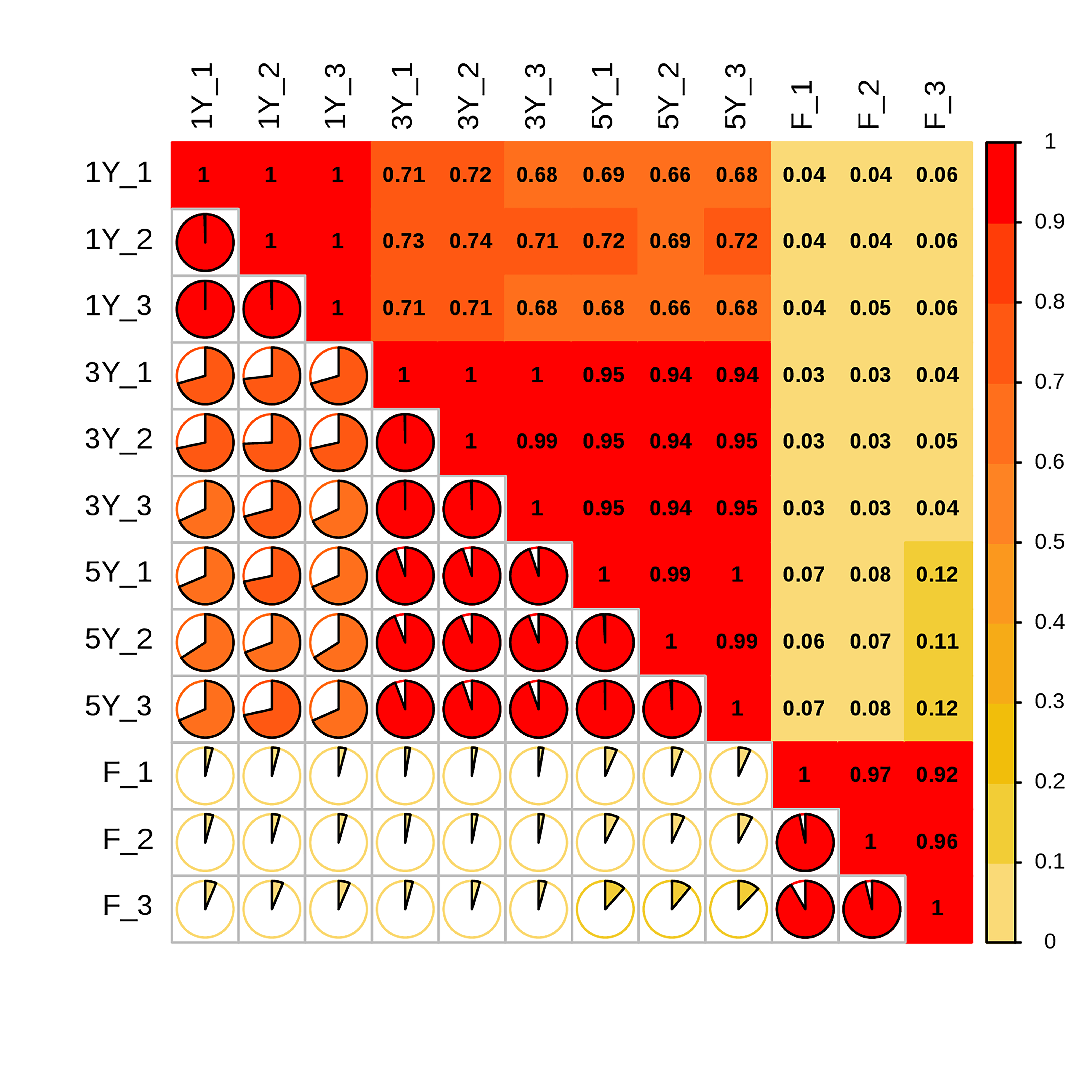

Supplement: Supplementary file 3 — Supplementary Material 3 [file 12864_2024_10608_MOESM3_ESM.png]

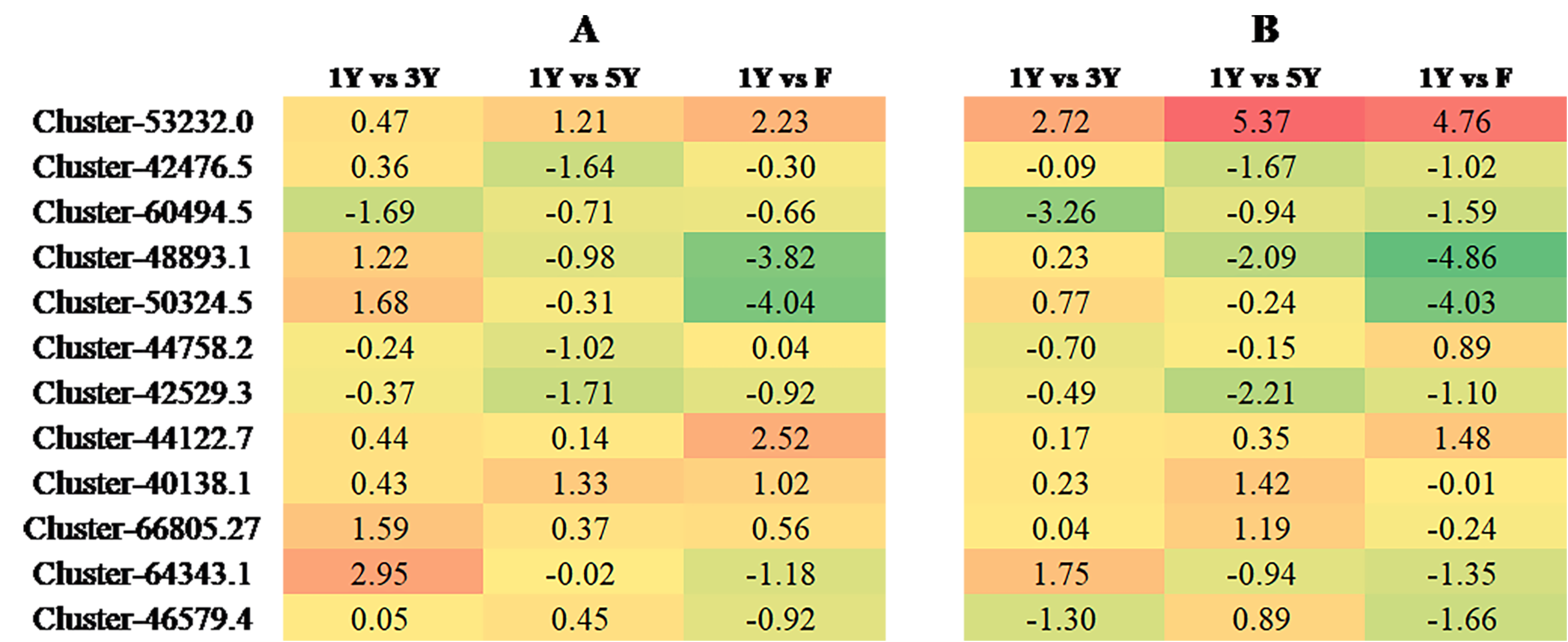

Supplement: Supplementary file 4 — Supplementary Material 4 [file 12864_2024_10608_MOESM4_ESM.png]

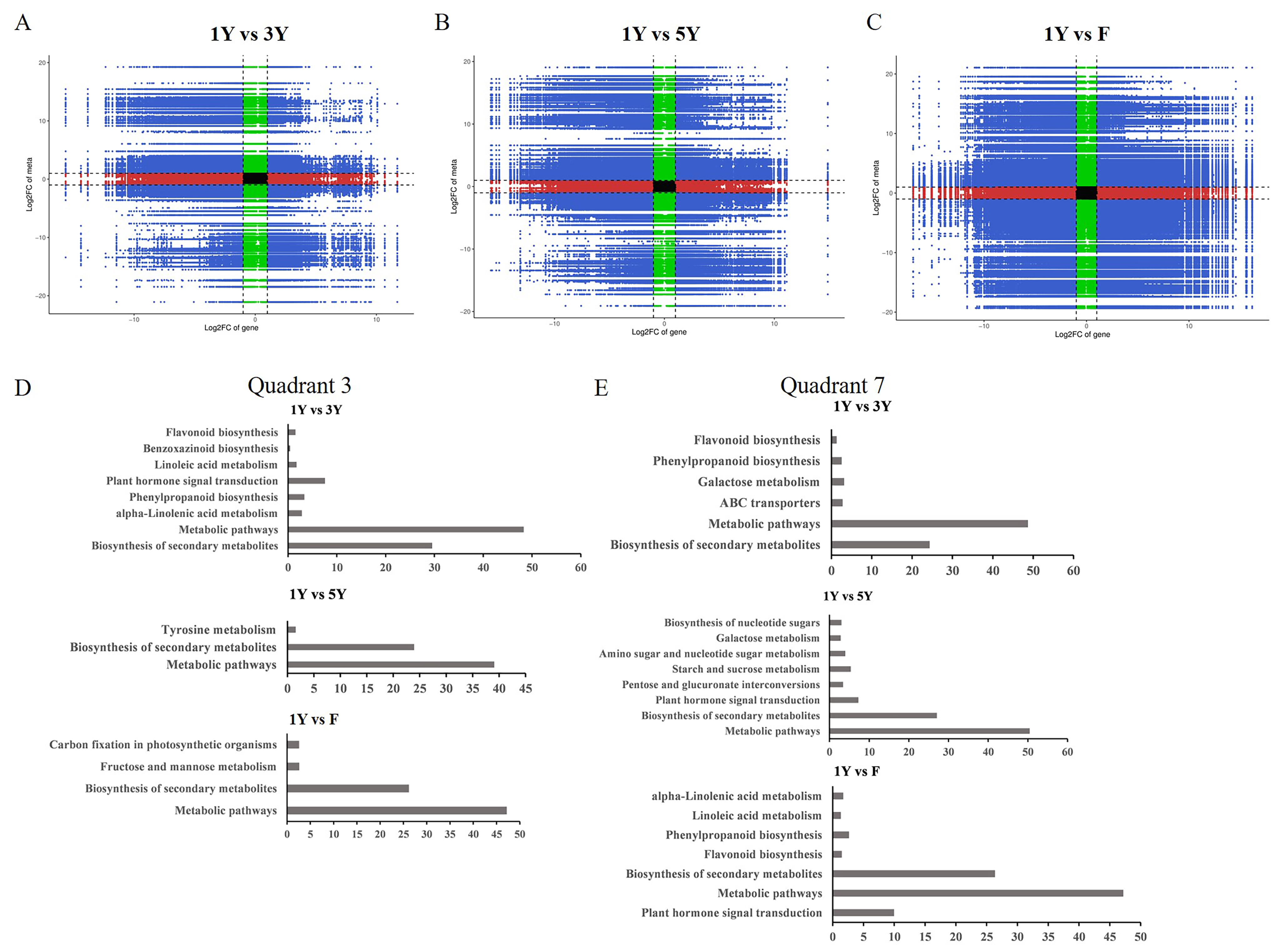

Supplement: Supplementary file 5 — Supplementary Material 5 [file 12864_2024_10608_MOESM5_ESM.jpg]
